# Lung Ultrasound in Critical Care and Emergency Medicine: Clinical Review

**DOI:** 10.3390/arm91030017

**Published:** 2023-05-17

**Authors:** Eduardo Rocca, Christian Zanza, Yaroslava Longhitano, Fabio Piccolella, Tatsiana Romenskaya, Fabrizio Racca, Gabriele Savioli, Angela Saviano, Andrea Piccioni, Silvia Mongodi

**Affiliations:** 1 Department of Translational Medicine, University of Eastern Piedmont, 28100 Novara, Italy; 2 Department of Anesthesia and Critical Care Medicine, AON SS. Antonio e Biagio e Cesare Arrigo H, 15121 Alessandria, Italy; 3 Department of Anesthesiology and Perioperative Medicine, University of Pittsburgh, Pittsburgh, PA 15260, USA; 4 Department of Anesthesia and Critical Care Medicine, AO Mauriziano Hospital, University of Turin, 10124 Turin, Italy; 5 Emergency Medicine and Surgery, IRCCS Fondazione Policlinico San Matteo, 27100 Pavia, Italy; 6 Department of Emergency Medicine, Policlinico Gemelli/IRCCS University of Catholic of Sacred Heart, 00168 Rome, Italy; 7 Department of Anesthesia and Intensive Care Medicine, Critical Care Unit-1, Fondazione IRCCS Policlinico S. Matteo, 27100 Pavia, Italy

**Keywords:** lung ultrasound, point-of-care ultrasound, pneumothorax, pleural effusion, lung aeration, acute respiratory failure, pneumonia

## Abstract

**Highlights:**

**What are the main findings?**

- There has been remarkable growth in lung ultrasound publications over the last decade. Given the large amount of new data available on this topic, a practical clinical review summarizing the most recent findings in this matter could help the physicians gain confidence in performing the technique.
- Following the recent ESICM consensus statement on critical care ultrasound, questions have been raised regarding the definitions of different skill levels. There are no clear criteria for differentiating basic skills from intermediate or advanced. In addition, the training path remains unclear.

**What is the implication of the main finding?**

- A practical, up-to-date approach to lung ultrasound in intensive care units and emergency departments. Supplemented by diagnostic and interventional flowcharts to guide its clinical application in everyday practice based on common clinical scenarios.
- In order to categorize the different levels of skill in lung ultrasound, we propose a new four-level classification which aims to describe, from basic to expert, the various competence that can be achieved.

**Abstract:**

Lung ultrasound has become a part of the daily examination of physicians working in intensive, sub-intensive, and general medical wards. The easy access to hand-held ultrasound machines in wards where they were not available in the past facilitated the widespread use of ultrasound, both for clinical examination and as a guide to procedures; among point-of-care ultrasound techniques, the lung ultrasound saw the greatest spread in the last decade. The COVID-19 pandemic has given a boost to the use of ultrasound since it allows to obtain a wide range of clinical information with a bedside, not harmful, repeatable examination that is reliable. This led to the remarkable growth of publications on lung ultrasounds. The first part of this narrative review aims to discuss basic aspects of lung ultrasounds, from the machine setting, probe choice, and standard examination to signs and semiotics for qualitative and quantitative lung ultrasound interpretation. The second part focuses on how to use lung ultrasound to answer specific clinical questions in critical care units and in emergency departments.

## 1. Introduction

Lung ultrasound (LUS) was considered unhelpful in the past due to the intrinsic impossibility of the ultrasound to penetrate the air; the only recognized indication was the assessment of pleural effusion [1]. Over the last few years, its usefulness for the clinical management of different diseases has been widely demonstrated [1,2]. Point-of-care ultrasonography may reduce the use of chest x-rays, computed tomography (CT) scans, and other radiation imaging techniques in the Intensive Care Unit (ICU) and in the Emergency Department (ED) [3,4,5]. The integration of bedside ultrasonography in the daily clinical activity of intensivists could reduce the risk of radiation exposure, need of patient transport, and hospital costs [6] and may redirect patient’s management [7]. A qualitative LUS approach is based on interpreting artifacts (A- and B-lines) and real images to distinguish between normal and pathological context. If a qualitative approach gives important information on the morphological assessment of the lung for the diagnosis, a quantitative approach allows us to extend the utility of the examination to lung monitoring [8]. The first section of this review aims to review how to perform a standard LUS examination and the basic LUS signs needed to interpret the images. The second section focuses on the ultrasound approach to different clinical scenarios frequently encountered in the daily clinical practice of physicians working in ED and ICU.

## 2. Basic Signs and Complete Ultrasound Examination

### 2.1. Machine Setting and Probes

**Figure 1:**
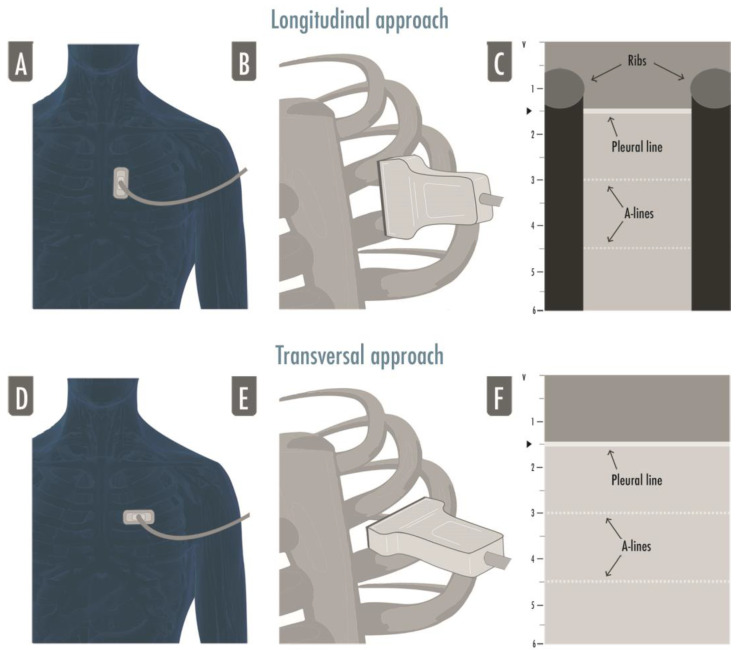
The figure illustrates both approaches to lung ultrasound: the longitudinal approach (**A**–**C**) and the transversal approach (**D**–**F**). In the longitudinal approach, the probe is aligned to the craniocaudal axis of the patient and perpendicular to the ribs’ axis (**A**,**B**), giving the characteristic ultrasonographic image of the two ribs and their shadows defining the pleural line in the middle, the so-called bat sign (**C**); in the transversal approach the probe is placed in the intercostal spaces, parallel to the ribs’ axis (**D**,**F**), so that a larger pleural section can be displayed without any rib’s shadows visualized (**F**).

Basic ultrasound machines can fit perfectly to perform LUS; modern machines usually have harmonics and artifact-erasing software that have to be deactivated to perform a proper LUS exam. In fact, most of the LUS signs are artifacts generated by the air–tissue interface and should not be erased by advanced software [2]. No specific probe is recommended to perform a general lung examination [8]. A micro-convex probe with a wide range of frequency can be useful, as well as a combination of a linear high-frequency probe and a convex/phased-array low-frequency one [2]. It depends on the clinical question that needs to be answered, on the setting in which ultrasonography is performed, and on the sonographer’s confidence. High frequency linear probes have a better definition of the superficial tissues (i.e., for the assessment of pleural line, pleural movements, and artifacts derived from the pleural line), and low frequency probes, such as the cardiac one, provide a better visualization of deeper findings (e.g., consolidations and effusions) and may be more useful for assessing the lung bases [2,8]. For a standard complete examination, we suggest starting with a linear probe in anterior fields and switch to a low frequency probe to examine the posterior regions. The “focus”, which is the depth where the ultrasound machine gives the maximum image resolution, usually indicated as a marker next to the centimetric depth scale of the ultrasound image, has to be placed as near as possible to the pleural line: LUS artifacts are generated from the pleural line, so a better definition of the pleural line allows for a better visualization of the artifacts. The depth has to be adjusted according to the anatomical characteristics of the patients, the lung region examined, and the probe used; usually a 6–8 cm deep image is adequate for the evaluation of anterior and lateral regions whether the posterior fields may require a deeper setting, mainly if consolidations/effusions are visualized. The probe’s orientation can be longitudinal (where the upper and lower ribs and the pleura are visualized forming what is referred to as the “bat sign”) or transversal (where the probe is positioned between two ribs, perfectly aligned to them, permitting the visualization of a larger part of pleura without the rib shadows) (Figure 1) [9].

### 2.2. LUS Examination

Two types of LUS examination can be identified: a comprehensive exam and a focused exam.

**Figure 2:**
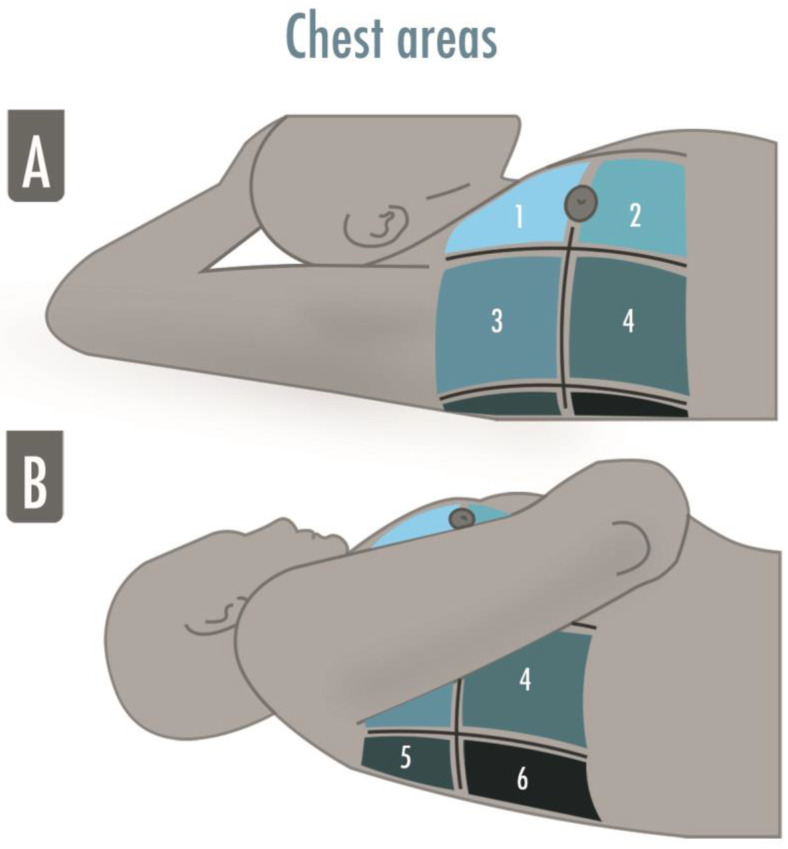
The illustration shows the six-areas division of a hemithorax in a supine patient for a comprehensive lung ultrasound examination. The anterior and the posterior axillary lines divide the hemithorax into three parts, which are finally divided into superior and inferior. The areas may be numbered from “1” to “6”, corresponding, respectively, to the anterior–superior and the inferior–posterior areas. (**A**) Zone 1 and 2 are superior and inferior anterior scans, zone 3 and 4 are superior and inferior lateral scans; (**B**) Zone 5 and 6 are superior and inferior posterior scans.

A comprehensive standard LUS examination includes twelve regions, six per side, and is indicated in the critically ill to assess and monitor pulmonary aeration similar to a complete and systematic evaluation: zone 1 and 2 are superior and inferior anterior scans, 3 and 4 are the lateral ones, and 5 and 6 are the posterior ones (Figure 2).

A focused LUS examination is simpler and wants to answer specific clinical questions, such as “Does my trauma patient have pneumothorax?” or “Did I completely drain the pleural effusion?”. In this case, the choice of probe and areas examined may vary according to the answer sought by the sonographer.

For instance, while performing extended focused assessment with sonography for trauma (eFAST), a physician may want to keep the same convex probe used to look for free fluid in the abdomen (it is also for a thoracic examination in order to save time) to rule out a significant pneumothorax in an emergency situation, only one anterior field per side will be sufficient to answer our clinical question.

### 2.3. Obtaining and Optimizing LUS Images

Bearing in mind that LUS artifacts derive from the tissue–air interface at the pleural line, it is mandatory to clearly detect the pleural line in order to avoid misinterpretation. The pleural line is usually located 0.5 cm below the ribs’ line, and it always corresponds to the parietal pleura whether the visceral one can be present or not [8]. Once the intercostal space is identified in the longitudinal scan, the tilting of the probe is useful to orient the ultrasound beam perfectly perpendicular to the pleura; the rocking helps in visualizing the pleural line as parallel to the probe footprint. These movements allow us to optimize the visualization of A-lines, a marker of a good quality image. Once the intercostal space is identified, a rotation centered on the pleura is useful to entirely visualize the pleural line while avoiding ribs’ shadows, switching from a longitudinal to a transversal scan.

### 2.4. Semeiotic

The main signs of LUS are artifacts generated by the high difference in acoustic impedance of subcutaneous tissues above the pleurae and air beneath them. They can be visualized in bidimensional images with the brightness-mode (B-mode). Additionally, the one-dimensional motion mode (M-mode) can be useful for fine motion assessment.

The main LUS signs are:

- Bat sign: the pleural line (bat’s body) is a horizontal hyperechoic line usually visualized in adult patients 0.5 cm below the ribs (bat’s wings) in a longitudinal approach; this is a basic landmark useful for proper identification of intercostal space and pleura. It is important, especially in those patients where it is difficult to identify the intercostal space as in case of subcutaneous emphysema or morbid obesity [9];
- A-lines: horizontal artifacts visualized as hyperechoic lines below the pleural line; repeated at a constant distance equal to the distance between the pleural line and the probe [10]; they are generated by the reverberation of the ultrasound beam between the pleura and the probe. The A-lines tell us there is air beneath the pleural line and correlate well with the high gas/volume ratio [8]; when associated with lung sliding, they correspond to normal lungs; otherwise, they can also be visualized in the case of hyperinflation and pneumothorax [2];
- B-lines: vertical artifacts originating from the pleural line, moving synchronously with it, erasing the A-lines and reaching the bottom of the screen. They are generated by increased density beneath the visceral pleura (altered air/tissue ratio) [11];
- Lung sliding: movement of the pleural line synchronous with tidal ventilation, it indicates that visceral and parietal pleura are in touch and regional ventilation is present [2];
- Seashore sign: straight lines above the pleural line and sandy pattern below the pleural line visualized in M-mode, confirm the lung sliding [2];
- Stratosphere sign: straight horizontal lines above and beneath the pleural line visualized in M-mode corresponding to an absence of pleural line movement, suggesting parietal and visceral pleura may not be in touch (i.e., pneumothorax), but also present in emphysematous bullae, pleural adherences, and severe hyperinflation;
- Lung Pulse: movement of the pleural line synchronous to the cardiac rhythm caused by the transmission of the heart beats; heart beats are always visible between breaths, but the sign is defined as a lung pulse only in the absence of lung sliding. It indicates that the pleurae are in touch, but regional ventilation is impaired (e.g., selective intubation, initial phase of atelectasis, pulmonary contusion, hyperinflation) [12,13];
- Lung Point: contact point between collapsed lung and pneumothorax air collection; a normal LUS pattern is visualized close to a motionless pleura; in M-mode, it could be visualized as an alternation between seashore and stratosphere sign, it represents the lateral edge of the intrapleural air layer [14].

LUS signs visualized in the case of consolidations and effusions:

- Shred sign: subpleural echo-poor images delimitated by irregular borders, indicating juxtapleural small consolidation [2];
- Tissue-like pattern: homogeneous texture of a lobe, similar to abdominal parenchyma, corresponds to a complete loss of aeration [15];
- Air bronchogram: hyperechoic intraparenchymal images visualized within a tissue-like pattern that corresponds to air trapped within the consolidation and that could be classified in absent, static (not patent airway), and dynamic (patent airways); the latter is then subclassified in linear/arborescent (specific for ventilator associated pneumonia) or punctiform (low specificity);
- Pleural effusions: hypo or anechoic space between the pleurae usually visualized in the most dependent areas of the chest. Its position could change in accordance with the patient’s posture, and the lung could be floating in it or compressed by it in a tissue-like pattern. Its echogenicity could help in differentiating the effusion’s type: transudative (i.e., homogeneously anechoic) or exudative (i.e., anechoic or homogeneously echogenic with internal echoes, fibrin strands, or septation).

### 2.5. Score for Lung Aeration Quantification

**Figure 3:**
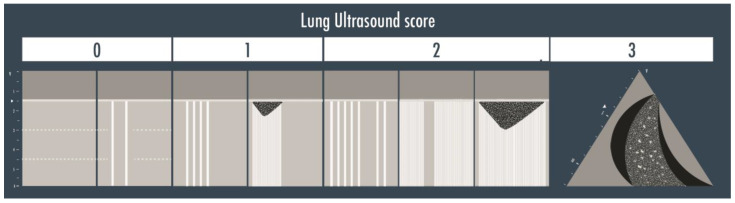
The lung ultrasound score is represented in the illustration above. Score 0 corresponds to the visualization of the A-lines only or with less than three B-lines per scan; score 1 is given if three or more well defined B-lines or coalescent B-lines/sub-pleural consolidations are visualized but they cover < 50% of the inspected zone; score 2 corresponds to the presence of multiple B-lines (coalescent or not) or sub-pleural consolidations occupying > 50% of the visualized pleura; score 3 is finally assigned when a clear tissue-like pattern is observed.

A twelve-zone examination, six per side, has been validated in the critically ill to assess and monitor pulmonary aeration for a complete and systematic evaluation (Figure 2) [16]. A score from 0 to 3 is given to each scan (score 0 = normal aeration with A-pattern or no more than two B-lines; score 1 = moderate loss of aeration with three or more well-spaced B-lines or coalescent B-lines/subpleural consolidation occupying < 50% of the pleural line; score 2 = severe loss of aeration with B-lines, coalescent or not, or a subpleural consolidation occupying clearly > 50% of the pleural line; score 3 = complete loss of aeration with consolidation, a tissue-like pattern). The global LUS score corresponds to the sum of regional scores and ranges between 0 and 36 points (Figure 3) [8].

## 3. Lung Ultrasound in Common Clinical Scenarios

### 3.1. Does My Patient Have a Pneumothorax?

**Figure 4:**
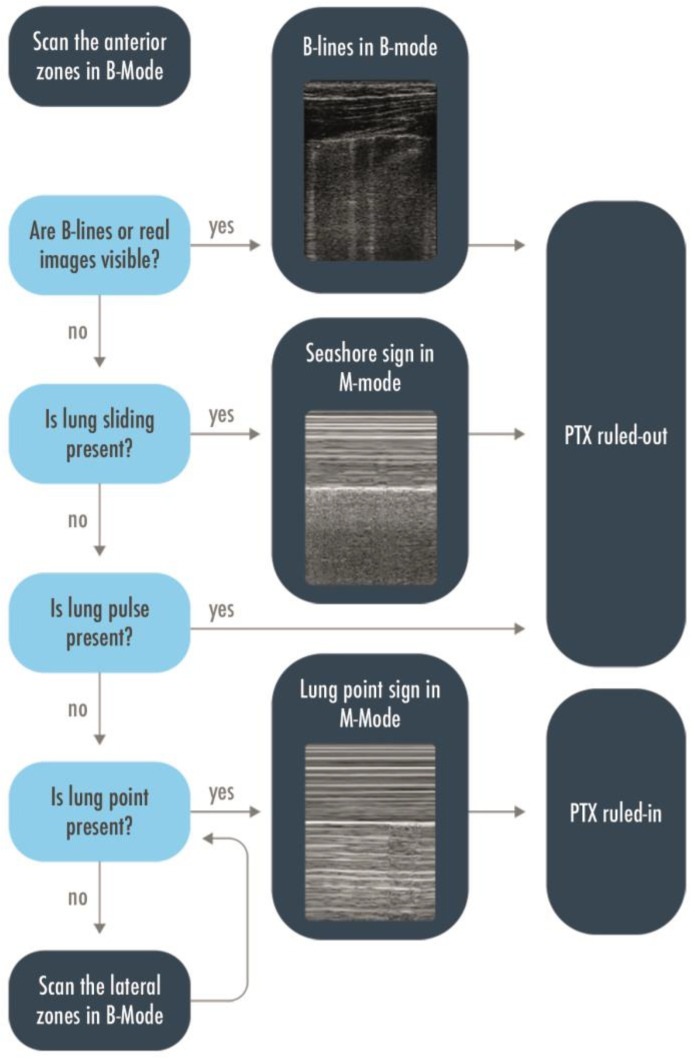
Flow-chart to rule in or rule out a pneumothorax. Scanning the anterior zones, detection of B-lines, real images, lung sliding, or lung pulse have to be found to rule out a pneumothorax. The only sign that could rule in pneumothorax is the lung point, and to find it, it may be necessary to extend the research to the lateral zones. PTX = Pneumothorax.

LUS may be used either in the ED or in the ICU to rule in or rule out a pneumothorax, to give the indication for a drainage and localize the site of insertion of the tube, and to semi-quantify the extension of the pneumothorax and monitor the residual presence of air after the drainage [17,18]. Misdiagnoses are frequent with anteroposterior chest X-ray (CXR), especially for an anterior pneumothorax, which cannot be easily visualized in supine patients. Computed tomography (CT) is the gold standard technique to confirm the diagnosis, but the patient must be transferred to the radiology service to get the exam, and it may take too long, in particular in unstable patients or hypertensive pneumothoraxes. LUS was proposed many years ago as a technique to rule out a pneumothorax in intubated patients [19], and its usefulness has been confirmed by further studies [11,19]. Either in a trauma patient or in a critically ill, mechanically ventilated patient, when a pneumothorax is suspected, the anterior areas of the thorax have to be evaluated first. Visualization of real images (consolidations/effusions) or B-lines, each representing a safe demonstration of the pleurae adhesion, permits us to rule out a pneumothorax with a negative predictive value of 100% [20,21]. After having correctly identified the pleural line, the lung sliding has to be looked for: lung sliding rules out a pneumothorax with a 100% negative predictive value. An M-mode could be used to help in its identification, when doubtful, thanks to its higher frame rate, and to objectify the motion of the pleura in a printable image. The seashore sign should be then visualized [13]. If the lung sliding is absent but the lung pulse is visualized, a pneumothorax is again ruled out with a 100% negative predictive value. In the absence of sliding and a lung pulse, the pneumothorax has to be suspected but could not be ruled in. This corresponds to the visualization of the static A-pattern, corresponding in M-mode to a stratosphere sign [21,22,23]. The visualization of a lung point confirms the presence of a pneumothorax with a 100% specificity. Moving the probe to the lateral areas of the thorax could help find this sign; in the case of a pneumothorax with a complete collapse of the lung, the lung point is not detectable and that is, partly, the cause of its low sensitivity [14,24,25]. A flowchart including the above-described sonographic signs has been proposed to rule out or rule in a pneumothorax (Figure 4) [13]. The exact distance between the chest wall and the collapsed lung is not quantifiable; in fact, a 1 mm thick air collection will generate the same signs of a centimetric one. However, the position where the lung point is detected on the chest wall corresponds to its surface extension and ideally to its thickness, considering that the more lateral the lung point is the greater the air collection. Indeed, LUS capability to estimate a pneumothorax volume close to the real one has been demonstrated to be much superior to the chest radiography compared to a volumetric CT in free air collections [26]. A lung point median to the mid axillary line indicates a 15% lung collapse and suggests a conservative management whether a lung point lateral to the mid axillary line represents a more relevant percentage of collapsed lung and, therefore, constitutes an indication for drainage [17]. This has been validated in trauma patients, so frequently young and previously healthy subjects with free air collection; a more cautious interpretation should be performed in mechanically ventilated patients, mainly with respiratory diseases potentially leading to non-free collections. In the expert recommendations recently published, ruling out a pneumothorax, identifying the lung point, integrating LUS with the clinical assessment to determine the indication for pneumothorax drainage, and identifying the location of the insertion of the tube are considered basic skills for the intensivist and are strongly recommended; the assessment of the topographic projection over the chest of the lung point and the semi-quantification of the pneumothorax extension are weakly recommended as basic skills [27].

### 3.2. Does My Patient Have a Pleural Effusion, and How Can We Estimate It?

Pleural effusion is visualized in B-mode as a hypo- or anechoic space between the pleurae; in case of low fluid viscosity, the lung is visualized floating in it, corresponding in M-mode to a sinusoidal movement called a “sinusoid sign” [28,29]. LUS is historically known to be a useful technique for the diagnosis of pleural effusion; it is more sensitive and more specific than CXR, allowing us to detect minimal effusions and to distinguish pleural effusion from consolidations, respectively [30]. An effusion as small as 5 mL can be detected with LUS with a 100% of sensitivity, whereas a minimum of 150 mL has to be present for detection with a CXR [31,32]. LUS enables us to quantify the effusion volume with an accuracy comparable to a CT scan, in a study of 36 patients hospitalized in a respiratory unit who underwent a chest CT scan and LUS, a strong correlation between the two was observed (intraclass correlation coefficient > 0.9) [33]. The echotexture of the collection may also help distinguish an exudative effusion, presenting with fibrin strands or septa, from a transudate, usually homogeneously anechoic [30,34,35].

Either in a decompensated patient entering to the ED or in a long-term critical care patient, LUS could be performed to evaluate, quantify, qualify, and monitor a pleural effusion. A micro-convex, convex, or phased array probe has to be used to obtain a better definition of deeper findings; the depth has to be set to 10–15 cm depending on anatomical characteristics and regions of interest, in this istance the posterior ones, with the patient sitting or supine. Because of the complex three-dimensional shape of the chest and of the different lung parenchymal status in the shape of pleural fluid, it is difficult to precisely assess the fluid volume, and there is still not a universally accepted method to determine it [36]. Multiple formulae have been evaluated in both positions, and the second formula proposed by Goecke for erect or seated patients [estimated volume in ml = (X + LDD) 70], assessed with a longitudinal approach, was shown to have a stronger correlation with the real volume in comparison with the formulae of Eibenberger or Balik applied to supine patients: where X = craniocaudal extent of the effusion at the dorsolateral chest wall; LDD = lung base to mid-diaphragm distance/subpulmonary height of the effusion in cm [37,38,39]. Even though, in critical care patients, where a proper seated position could not be achieved and free pleural fluid gravitates posteriorly, forming a sickle-shaped lamella, a transversal approach allows us to measure the maximum distance between the pulmonary surface and chest wall and, therefore, to identify the maximum volume by the proposed Balik’s formula (estimated volume in mL = 20X) or Eibenberger’s one (estimated volume in mL = 47.6X − 837), where X = maximum perpendicular distance between the pulmonary surface and the chest wall at maximal inspiration in mm [36]. Due to its lack of linearity, Eidenberger’s formula is limited to cases where a large amount of fluid is present. In experts’ recommendations, the identification of a pleural effusion, its volume estimation, and distinction of internal echoes, which could help in differentiating complicated effusions (e.g., exudates, empyema, hemorrhage), are considered basic skills for an intensivist performing LUS in an intensive care setting. Likewise, indication for a chest drain insertion, where to place it, monitoring of its effectiveness and complications are recommended [27,39].

### 3.3. Why Is My Patient Hypoxemic?

**Figure 5:**
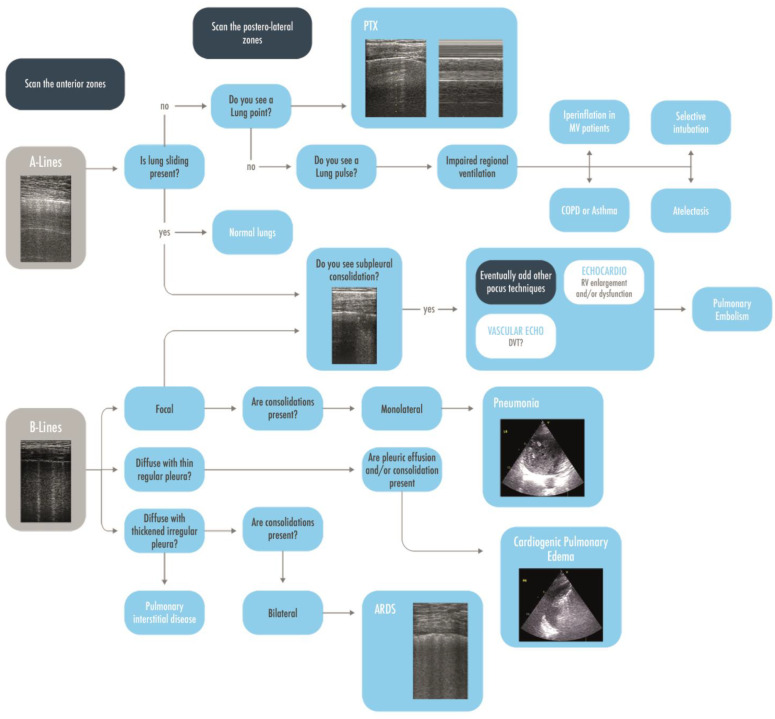
A brief practical ultrasonographic diagnostic approach to the hypoxemic patient, starting from the visualization of A-lines and B-lines in the anterior areas and, thereafter, evaluating other more specific signs and the posterolateral zones. ARDS = Acute Respiratory Distress Syndrome; COPD = Chronic Obstructive Pulmonary Disease; CPE = Cardiac Pulmonary Edema; DVT = Deep Venous Thrombosis; MV = Mechanically Ventilated; PEEP = Post End Expiratory Pressure; RV = Right Ventricle.

LUS is sensitive to changes in lung aeration and density. Increased lung water, deflation or a combination of the two phenomena modify the images visualized and give relevant clinical information [40]. Based on these concepts, three patterns have been identified to evaluate and monitor the degree of pulmonary aeration and density: A-pattern is visualized when the proportion between air and fluid is preserved (e.g., normal parenchyma) or air is increased (e.g., pulmonary emphysema); B-pattern is defined by at least 3 B-lines in an intercostal space (ranging from regularly spaced B-lines to shining and merged ones of the white lung) and is referred to as an increased lung density; Consolidated pattern when no air is present, either caused by a complete deaeration or because alveoli are completely filled with fluid [41]. Beginning with the evaluation of the anterior fields and then extending the examination to lateral and posterior ones, an A-pattern orients to a normal aeration; if lung sliding is absent, a pneumothorax is suspected and eventually confirmed by the visualization of the lung point. If, instead, a lung pulse is visualized, an impaired regional ventilation must be suspected, orienting to an acute decompensation of chronic obstructive pulmonary disease (COPD), asthma, initial phase of atelectasis (e.g., selective intubation), and hyperinflation in mechanical ventilated patients. If a normal lung sliding is visualized associated with a subpleural consolidation in a hypoxemic patient, this should remind the physician of a pulmonary embolism (PE) and lead to perform focused cardiac ultrasound and compressive vascular ultrasound, looking for signs of right ventricular enlargement and/or dysfunction and deep venous thrombosis [2]. Instead, when a B-pattern is visualized, it has to be differentiated on the basis of its distribution: if a focal B-pattern with subpleural consolidations and/or mono-lateral consolidations are present, a pulmonary embolism or a pneumonia has to be suspected. A diffuse B-pattern distribution (i.e., at least two regions per side) with normal sliding, thin regular pleura, and eventual bilateral posterior consolidations and/or bilateral pleural effusions orients to cardiogenic pulmonary edema; whereas if the sliding is reduced, the pleura is thickened and irregular, and posterior consolidations with no effusions are visualized, the diagnostic hypothesis shifts towards interstitial pneumonia/acute respiratory distress syndrome (ARDS) (Figure 5) [42,43,44]. An ultrasound-aided approach to patients with respiratory failure in ED led to an increased number of early correct diagnoses, early correct treatments, and finally, to a better use of advanced imaging as a CT scan [45]. The international scientific societies nowadays recommend using LUS for the evaluation and grading of pulmonary edema during heart failure [46,47] and of pulmonary fibrosis or sarcoidosis [48]. Integration of LUS in the clinical examination to evaluate respiratory failure is nowadays strongly recommended as a basic skill for an intensivist, as well as the recognition of the different patterns of increased lung density (e.g., B-pattern, lung consolidation). A meta-analysis of eleven studies, including 1232 patients, evaluated the diagnostic accuracy of LUS in critically ill patients with acute respiratory failure (ARF) and found an overall pooled sensitivity and specificity of 92% and 98%, respectively [49]. In the specific context of pulmonary embolism, a combination of A-pattern with deep venous thrombosis and subpleural consolidations (corresponding to pulmonary infarctions) has been demonstrated to be 90% sensitive and 86% specific in the ED [50]. However, it has to be kept in mind that ultrasound is not supposed to replace a clinical examination, which can be superior in the diagnosis of respiratory pathologies that have no specific ultrasound signs, such as asthma and COPD decompensation [51,52]. Automation with modern softwares analysing grey-scale texture of the pleural ultrasound images has furthermore been proposed to facilitate the interpretation of LUS in the critically ill (e.g., acute respiratory distress syndrome and cardiogenic pulmonary edema), but more trials are needed to confirm its superiority to the visual analysis of an expert, which remains so far the gold standard [52].

### 3.4. Does My Patient Have Ventilator-Associated Pneumonia (VAP)?

A VAP is suspected in patients under mechanical ventilation for more than 48 h with a combination of clinical indicators as the presence of lung opacity, impaired gas exchanges, fever/hypothermia, hyper-/hypo-leukocytosis, and increased and/or purulent secretions. The recommended gold-standard for the diagnosis of VAP is a quantitative microbiological analysis of a lower respiratory tract sample [53].

However, this may require 24 to 48 h for preliminary and definitive results, respectively, thus potentially leading to either a delayed introduction or an extended use of antibiotics. A bedside specific diagnostic tool would be of help; however, CXR opacities in ICU patients showed very low specificity for VAP [54,55] since it was present in most of the ICU patients [56]. No specific CT scan signs have been described, either [57]. 

**Figure 6:**
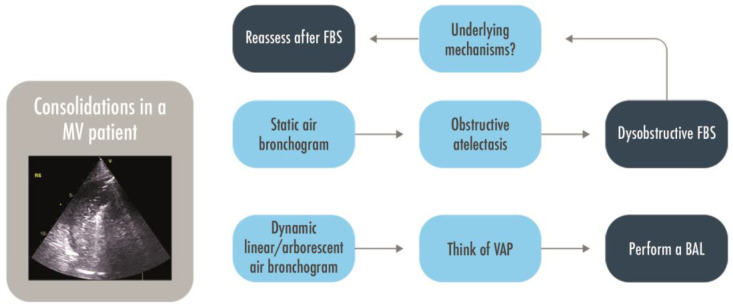
A decisional approach to pulmonary consolidations in intubated patients. BAL = Bronchoalveolar lavage; FBS = Fiber-bronchoscopy; MV = Mechanically Ventilated; VAP = Ventilator Associated Pneumonia.

LUS is a valid alternative for an early diagnosis of pneumonia in adults in an emergency setting [51,58] where the visualization of a consolidated lobe is highly specific for the diagnosis. In intensive care patients under mechanical ventilation, where more intricated mechanisms may lead to a complete loss of aeration, mainly in the posterior fields, the presence of a tissue-like pattern is highly non-specific for ventilator-associated pneumonia; in this context, the interpretation of the air bronchogram is an additional value. A dynamic linear/arborescent air bronchogram within a consolidation has been demonstrated to be highly specific for a VAP [59,60]. A dynamic air bronchogram rules out obstructive atelectasis, which are instead characterized by a static bronchogram (in the initial phase) or by the absence of any air bronchograms (no air is present neither in the small airways) [61]. From these concepts, a clinical decision could be made: a consolidation with a static air bronchogram or without it suggests a nonpatent airway and drives to the indication for a disobstructive fiber-bronchoscopy (Figure 6) [2,59]. In a clinical trial including 80 mechanically ventilated patients in an ICU, the diagnostic accuracy of LUS, CXR, and clinical pulmonary infection score (CPIS) compared to the CT scan as a gold standard was evaluated. LUS had high sensitivity and specificity (91.67% and 100%, respectively), as well as high positive and negative predictive value (100% and 96%, respectively); it performed significantly better than CXR (*p* < 0.001) and a CPIS > 6 (*p* < 0.001) [62]. A color Doppler is appliable on a consolidated lung to evaluate the vascularization. The detection of a lung region with a complete loss of aeration and good perfusion is an indicator of intrapulmonary shunt in consolidated lobes, contributing to hypoxemia. This evaluation remains although only qualitative [15]. LUS color Doppler with an evaluation of intrapulmonary flow and dynamic air bronchograms has been recently seen to be the most frequent signs in veno–arterial extracorporeal membrane oxygenation patients with hospital-acquired pneumonia, with a lung ultrasound simplified clinical pulmonary score leading to better results than the classical CPIS, bioclinical parameters, or CXR [63]. LUS has also been confirmed to be a valuable tool for the bedside identification of pulmonary overinfections in COVID-19 patients already admitted to ICUs for pneumonia and ARDS. In a retrospective observational study, dynamic linear/arborescent air bronchograms within lobar/hemilobar consolidations were demonstrated to have a very high specificity [60]. In patients monitored by a daily assessment of a lung ultrasound score, an increase in the LUS score should raise the doubt of an overinfection [4,64]. The quantitative evaluation of a lung ultrasound for the assessment of lung aeration, however, has not been considered as a basic skill by the expert recommendations of the European Society of Intensive Care Medicine (ESICM) [27].

### 3.5. Does My Patient Need PEEP or Prone Position?

**Figure 7:**
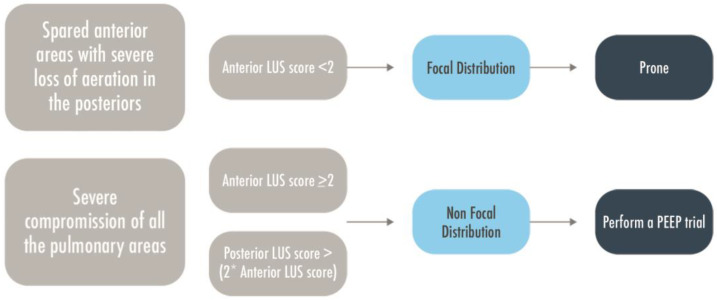
A brief flow-chart to predict patient’s responsiveness to PEEP or prone position. LUS = Lung Ultrasound; MV = Mechanically Ventilated; PEEP = Positive End Expiratory Pressure.

A personalized approach based on lung morphology and the identification of focal and diffuse patterns seemed not superior in the Live study to PEEP-FiO_2_ table in terms of survival of ARDS patients [65]. However, in this study the morphologic assessment was mainly performed with CXR, with a high percentage of misclassified patients that showed a severely increased mortality. If limiting the analysis to those correctly classified, the personalized approach determined a significant improvement in the survival rate. This underlines the importance of having a reliable method for the classification of lung morphology, both to allow personalization of ventilation strategy and to avoid misclassification, which negatively affects patients’ survival [66]. The LUS score has been proposed to classify lung morphology in focal and diffuse for invasively ventilated ARDS patients; when compared to the gold standard (i.e., CT scan), the anterior LUS regions showed to be the most discriminant between focal and non-focal morphology with an accuracy moderately increased with the integration of lateral and posterior findings [67]. Patients with a focal distribution, severe loss of aeration in the posterior areas, and spared anterior ones are usually prone-responders [68]. On the contrary, patients with diffuse distribution and compromission of all the fields, including the anterior ones are usually PEEP-responders (Figure 7) [69,70]. The LUS’ capability to predict the intensity of oxygenation response resulting from the prone position in severe or moderate ARDS was not confirmed by a prospective multi-center study; however, the overall responsiveness to pronation is normally not quantified by PaO_2_/FiO_2_ variations only. Moreover, the patients showing more significant improvement in LUS score after prone positioning were those with focal morphology [71]. The application of LUS to decide the ventilatory strategy and which is the best way to distinguish focal and non-focal patterns are still matters of research.

### 3.6. Does My Patient Risk Extubation Failure?

LUS also allows us to identify patients at risk of extubation failure when performed at the end of a spontaneous breathing test; LUS score is affected by any change in lung aeration and is, therefore, a common final pathway of different mechanisms leading to a lung loss of aeration during the weaning phase. It not only identifies the weaning induced pulmonary edema, but also the derecruitment due to unsolved lung diseases or inadequate respiratory muscle strength, making LUS a valuable screening tool for the patients at risk of extubation failure [72,73,74,75,76]. A global LUS score ≥ 17 or an antero–lateral LUS score ≥ 5 at the end of a spontaneous breathing trial indicate a high probability of a post-extubation need for respiratory support, suggesting further diagnosis of the underlying disease, eventually using other ultrasound techniques, such as a cardiac and diaphragm ultrasound, and to optimize the patient’s condition before a new weaning attempt [77]. The sensitivity and specificity observed were ≤ 90 % either in detecting weaning-induced pulmonary oedema [73] or in predicting postoperative ventilatory support [77]. The use of LUS as a guide for a ventilatory strategy was considered an advanced skill by the European team of ultrasound experts [27].

### 3.7. Does My Patient Have COVID-19?

Following the pandemic of COVID-19, the use of LUS boosted in the clinical practice and also in wards or medical structures where it was previously uncommon or not feasible. Physicians brought the LUS from the medical service to outpatient settings using portable devices [78,79]. The LUS was found to be an accurate tool to quickly diagnose, triage, and monitor patients with COVID-19 pneumonia [80]. In an Italian survey proposed to anesthesiologists and intensive care physicians after the first wave of the COVID-19 pandemic, the results showed that the LUS was extensively used during the first phase of the pandemic, and then, its adoption increased further [81]. Performing LUS in COVID-19 patients mostly revealed a pattern of diffuse interstitial lung syndrome with thickening of the pleural line, presence of pleural abnormalities, multiple or confluent bilateral B lines with spared areas and without a cranio-caudal distribution gradient, and peripheral consolidations [79]. Even though many general ultrasonographic findings have been described in these patients, no signs were specific or pathognomonic to COVID-19 pneumonia [82], making a differential diagnosis with other viral pneumonia difficult [79]. Indeed, in the setting of an ED, when combined with the medical history of the patient and a high clinical probability in a pandemic context, the presence of bilateral B-lines was associated with a higher positive likelihood ratio of a COVID-19 diagnosis, as confirmed by a positive RT-PCR test, whether patients with low clinical probability and no B-lines had a low negative likelihood ratio to have positive RT-PCR [83]. Despite the good association described, in patients with other comorbidities (e.g., acute cardiogenic pulmonary edema, chronic interstitial lung disease, etc.) presenting with acute respiratory failure, the interstitial syndrome identified by LUS could be difficult to be attributed to COVID-19 pneumonia rather than to a decompensated previous illness [80]. A recently published systematic review including 66 articles with a pool of 4687 patients observed that the most frequent ultrasound findings in COVID-19 patients were B-lines and pleural abnormalities and confirmed the association between a high LUS score at admission and unfavourable outcomes [84,85,86]. In a retrospective study including 93 patients with a suspected COVID-19 infection admitted to the ED, LUS was found to have sensitivity and negative predictive value (93.3% and 94.1%, respectively) similar to a CT but, instead, a much lower specificity, positive predictive value, and accuracy (21.3%, 19.2%, and 33.3%, respectively) [87]. Another retrospective study conducted in an ED of a tertiary care center confirmed its high negative predictive value compared to PCR results (89.2%) and observed a higher specificity (72.7%) but a lower sensitivity (70.6%) [88]. An approach based on ultrasound patterns was proposed to improve the ED triage of COVID-19 patients (A-low probability, B-Pathological finding on LUS, C-Intermediate probability, D-High probability) [89]. A good correlation between a chest CT scan and LUS score or hypoxemia severity has been observed in multiple studies [90,91]. Meanwhile, no significant correlation was observed between a quantitative assessment of lung damage by a lung ultrasound (lung ultrasound Zaragoza LUZ-score) and X-ray findings (Shalekamp score), and the ultrasound was found to be superior in assessing the outcome of COVID-19 patients during their hospital stay, especially if associated with a clinical prediction mode [92,93]. An evaluation of the LUS score in the ED could also assist physicians in the prognostic stratification [94]. A high LUS score on admission was in fact associated with the need for transfer to ICU, helping in deciding a patient’s destination and in predicting non-invasive ventilation failure and a need for invasive mechanical ventilation [95,96]. Finally, several complications could occur in a COVID-19 patient, limiting the diagnostic performance of LUS and making it necessary to consider laboratory findings and previous medical history [97]. For instance, in the case of a patient with a COVID-19 pneumonia and concomitant pulmonary embolism, of which the incidence has been largely described in this population [98,99], distinguishing the pleural signs related to each condition might be difficult. New perspectives could be opened by contrast enhanced ultrasound (CEUS), which could help in detecting peripheral areas of low perfusion and/or infarction in such patients [100,101]. However, a CT pulmonary angiography should then be considered as a first choice [102].

## 4. Discussion

**Figure 8:**
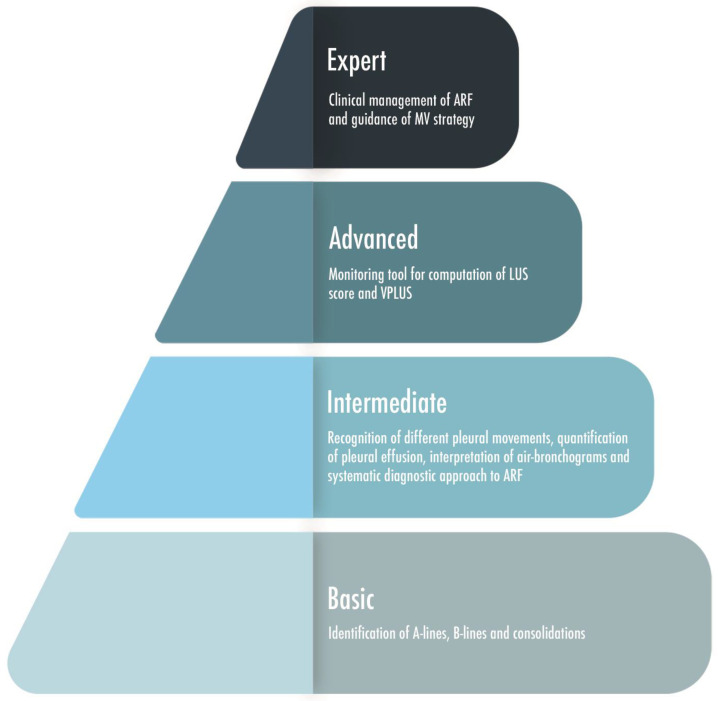
A graphic representation of four level steps of skills-knowledges based on the expertise of the physician performing lung ultrasound. ARF = Acute Respiratory Failure; LUS = Lung Ultrasound; MV = Mechanical Ventilation; VPLUS = ventilator-associated pneumonia lung ultrasound score.

After the recently published consensus statement on the critical care ultrasound of the ESICM [27], an issue has been raised about the definitions of basic and advanced skills [103]. Pleural effusion is easy to identify, but recognizing the etiology and determining the volume can be more challenging. Similarly, B-lines are easy to recognize, but their etiology and clinical interpretation could be difficult to assess. An echo-texture of a lung consolidation is easily identified, but distinguishing between atelectasis and pneumonia would require the sonographer to be capable of recognizing dynamic air bronchograms and to interpret the color Doppler, which probably are at least intermediate skills [103]. Concern has been created, indeed, for the lack of clearly defined criteria in the scientific literature for defining basic lung ultrasonographic skills and distinguishing them from the advanced ones, especially in comparison with the cardiac ultrasound in the critical care setting, which has more literature in support and well-defined steps of knowledge [104]. So far, the only recognized advanced skill is the computation of the LUS score, which clearly requires a dedicated training [105]. A four-step definition of LUS skills, classified on the basis of the items that determine if a particular skill is assessed as basic or advance, has been proposed [103]. The incidence of the considered disease, the easiness to acquire the images and to interpret it, and finally the capability of answering to relevant clinical questions have been evaluated and, based on these, a suggested number of training scans has been proposed as the minimum to acquire specific LUS skills. If 10 trained LUS are enough to rule out a pneumothorax, 60 trained exams are needed to learn how to differentiate between atelectasis and pneumonia, and 80 exams are instead requested to acquire the competences to rule in a pneumothorax. An international study, willing to standardize the LUS training and conducted in 10 ICUs, among resident and staff working in intensive care units, anesthesiology services, emergency medicine, and internal medicine wards, concluded that 25 transthoracic ultrasound examinations supervised by an expert provide the basic skills for diagnosing normal lung aeration, interstitial–alveolar syndrome, and consolidation in emergency and critically ill patients [106]. Four-level steps of knowledge may be hypothesized: the basic level includes a simple identification of A-lines, B-lines, and consolidations [88]; an intermediate level may include pleural movement interpretation (sliding, lung pulse, lung point) and additional skills as pleural effusion quantification, air-bronchogram interpretation, and systematic diagnostic approach to acute respiratory failure; the advanced knowledges may include a quantitative lung ultrasound as a monitoring tool (computation of lung ultrasound score and of ventilator-associated pneumonia lung ultrasound score); and the expert level includes qualitative and quantitative LUS in the clinical management of acute respiratory failure patients and as a guidance to mechanical ventilation strategy (Figure 8). Research will have to confirm this hypothesis. How to reach an adequate training, so far missing [81,107], is also a matter of concern.

## 5. Limitation

Many factors could influence LUS reproducibility, leading to a misinterpretation. As an ultrasound technique, LUS is operator-dependent [108]. Despite this, the inter-operator agreement observed assessing lung aeration is still excellent [16,109]. Artifacts are determined by the relationship between the curve of the probe used and the curve of the pulmonary surface examined. The use of different kinds of probes with various curvatures, frequencies, and gain compensation could lead to a lack of homogeneity between images of the same scan [110]. A deep insight into ultrasound physics and the nature of artifacts is then required to the operator for correctly setting the machine and performing the examination without getting the wrong conclusions [111]. Another concept that has to be taken into account is that the pleural surface visible with lung ultrasound is only the 70% of the total, whereas the rest is covered by the thoracic cage [112]. Finally, the learning curve could be fast at the first appearance, with 5 min of online training for learning how to rule-out a PNX [113] and a few hours of a theoretical–practical course needed to correctly detect a pleural effusion [114], but a longer path is needed to acquire more in-depth competences. A consensus regarding the training needed to reach a specific level of competence in lung ultrasound is lacking [115]. Taking into consideration the limits mentioned above, LUS has to be performed systematically in order to maximize its reliability [41].

## 6. Conclusions

LUS is reaching a leading role for the diagnosis and monitoring of respiratory diseases in different clinical settings, either in intensive care units or in sub-intensive and general wards. The contemporary ease of access to the ultrasound machines has certainly boosted its applicability in any context. It was finally demonstrated that LUS has an impact on clinical decision-making by changing the diagnostic process and patient’s management [116]. The introduction of the quantitative approach has further extended the field of its clinical application. Concerns have been raised nowadays about the definition of basic and advanced skills, and learning paths to the acquisition of the skills have to be better defined. LUS has the potential to reach a wider field of applicability and higher reliability, but well-designed trials are still necessary to clarify these gaps.

## Data Availability

Not applicable.

## Abbreviations

ARDS	Acute Respiratory Distress Syndrome
ARF	Acute Respiratory Failure
CAP	Community Acquired Pneumonia
CEUS	Contrast Enhanced Ultrasound
COPD	Chronic Obstructive Pulmonary Disease
CPE	Cardiogenic Pulmonary Edema
CPIS	Clinical pulmonary infection score
CT	Computed Tomography
CXR	Chest X-ray
DE	Diaphragmatic Excursion
ED	Emergency department
eFAST	Extended Focused Assessment with Sonography in Trauma
ESICM	European Society of Intensive Care Medicine
ICU	Intensive Care Unit
LP	Lung Point
LUS	Lung UltraSound
PE	Pulmonary Embolism
PEEP	Positive End Expiratory Pressure
TGC	Time Gain Compensation
US	Ultrasound
VAP	Ventilator Associated Pneumonia
